# Combination of Triple Therapy and Chronic PPI Use May Decrease Risk of Colonic Adenomatous Polyps in *Helicobacter pylori* Infection

**DOI:** 10.1155/2015/638547

**Published:** 2015-05-12

**Authors:** Rina Zuniga, Josef Bautista, Katherine Sapra, Keith Westerfield, Susan Williams, Alexander M. Sy

**Affiliations:** ^1^Department of Medicine, New York Medical College-Metropolitan Hospital Center, New York, NY 10029, USA; ^2^Institute for Psychoanalytic Training and Research, New York, NY 10025, USA; ^3^Mailman School of Public Health, Columbia University, New York, NY 10032, USA; ^4^Division of Gastroenterology, New York Medical College-Metropolitan Hospital Center, New York, NY 10029, USA

## Abstract

*Aim*. The aim of the paper is to determine association between *H. pylori* and colonic adenomatous polyps and to explore whether treatment or chronic PPI use can mitigate this risk. *Methods*. This case-control study included 943 patients who had *H. pylori* testing and underwent colonoscopy. Presence of polyps was the outcome of interest, whereas age, sex, race, *H. pylori* infection, triple therapy, and chronic PPI use were independent variables. Multivariate regression analysis was used to calculate odds ratios at 95% confidence intervals. This study was approved by the New York Medical College Institutional Review Board. *Results*. *H. pylori* was associated with increased odds of colonic adenomatous polyps (adjusted OR 1.43, 95% CI 1.04–1.77), with stronger association among patients older than 50 (OR 1.65, 95% CI 1.18–2.33). Triple therapy (OR 0.69, 95% CI 0.44–1.07) or chronic PPI use (OR 0.69, 95% CI 0.43–1.09) decreased odds of polyp formation. Analysis revealed a statistically significant reduction in patients who received both triple therapy and chronic PPI, lowering the odds by 60% (adjusted OR 0.43, 95% CI 0.27–0.67). *Conclusion*. There is increased risk of colonic adenomatous polyps among *H. pylori*-infected patients. Triple therapy or chronic PPI use may mitigate this risk, with further reduction when these two interventions are combined.

## 1. Introduction

*Helicobacter pylori (H. pylori)* is a bacterium which is indigenous to humans and it is estimated that at least half of the world's population is infected with it [1]. It is well adapted to exist in the stomach throughout the lifetime of its host and to incite a chronic inflammatory state during this period. In the United States, African Americans and Hispanics have been found to have high predilection to this infection compared to Caucasians. Males are equally affected as females [2]. It has been recognized by the International Agency for Research on Cancer as a class I human carcinogen due to its ability to induce gastric adenocarcinoma. It is firmly established that it can also cause extragastric disease, as the bacteria has already been identified in the hepatobiliary system, in the intestines and in feces [3]. Previous investigations linking* H. pylori* to extragastric cancer have reported finding* Helicobacter* DNA in 52.6% of hepatobiliary cancer cases [4], prompting investigators to explore the hypothesis that* H. pylori* may also be associated with intestinal polypoid structures and/or colorectal carcinoma. The pathophysiology was thought to be direct or local carcinogenic effect on colonic epithelia by the bacteria or through stimulation of hypergastrinemia. The presence of* H. pylori* has been linked with a prolonged and excessive release of gastrin [5], a hormone shown to promote the growth of colon cancer cells in culture [4] and to exert a remote trophic effect on colonic mucosa [4].

Colorectal cancer is the second leading cause of cancer-related deaths in the United States when both sexes are combined. The American Cancer Society estimates it to cause about 49,700 deaths during 2015 [6].

Prior studies done to determine the association between* H. pylori* and colonic neoplasia have shown conflicting results. While a number of studies have shown direct correlation between* H. pylori* carriage and colorectal cancer risk [1, 4, 7–11], others failed to show this correlation [3, 5, 12–15]. In this study, we investigated the potential relationship between* H. pylori* infection and the risk of developing colonic neoplasia. Furthermore, we aimed at specifically exploring how* H. pylori* treatment with triple therapy and chronic proton pump inhibitor (PPI) use can influence the development or regression of colonic adenomatous polyps.

## 2. Methodology

### 2.1. Study Population

This is a single-center retrospective case-control analysis of the risk of developing colonic adenomatous polyps among adults with* H. pylori* infection. Review of electronic medical records of Metropolitan Hospital Center in New York was done during the period from January 1, 2010, to December 31, 2012. Inclusion criteria included adult patients who underwent screening colonoscopy and had an esophagogastroduodenoscopy (EGD) or antigen detection for* H. pylori* prior to the colonoscopy. We excluded patients who (a) had proven colorectal carcinoma, (b) had high risk of developing colorectal carcinoma (i.e., diagnosed cases of inflammatory bowel syndrome, positive family history of polypoid syndromes, and prior history of carcinoma), and (c) underwent incomplete colonoscopy or had inadequate preparation for colonoscopy. In subjects who underwent multiple EGDs and colonoscopies, we included the index colonoscopy in the analysis. After implementation of our exclusion criteria, we included 943 patients in the final analysis.

### 2.2. Colonoscopy and Definition of Outcomes

Excised polyps were sent to the Department of Pathology for cytopathologic identification. Colonoscopy result was recorded as being adenomatous polyp positive, hyperplastic polyp, or no polyp. Cases were defined as the presence of biopsy-proven adenomatous polyps, whereas controls were defined as the absence of polyps or presence of hyperplastic polyps. Adenomatous polyps include tubular, tubulovillous, or villous polyps. Data regarding polyp location and number were also obtained. We categorized location as either right-sided for polyps located in the cecum and the proximal two-thirds of the transverse colon, left-sided for polyps located in the distal third of the transverse colon until the rectum, or both for multiple polyps located in both left and right sides.

### 2.3. *H. Pylori* Status as Exposure

Exposure was defined as the presence of* H. pylori* infection prior to colonoscopy. Determination of* H. pylori* status was made either by the rapid urease test or culture of tissue sample obtained through EGD or by* H. pylori* antigen detection in serum or stool samples. Data regarding* H. pylori* treatment with triple therapy and chronic PPI use were gathered. Completion of the course of the triple therapy (two weeks of Amoxicillin and Clarithromycin and six weeks of PPI) was the working definition of treatment in our data collection. Of note, no patients received quadruple therapy in our sample and there were only five patients who received salvage treatment for* H. pylori*. Chronic PPI use, a variable independent of the* H. pylori* treatment, was defined as use of any PPI for at least six months prior to or up to the time of the index colonoscopy. Data on use of H_2_ blockers, sucralfate, or aluminum-magnesium hydroxide were not gathered nor included in the analysis.

### 2.4. Statistical Analysis

Baseline characteristics were compared using Chi-square statistics for all categorical variables with *n* more than five. Continuous variables were assessed using Wilcoxon Rank-sum or *t*-test statistics. A two-sided *p* value < 0.05 was considered significant and included in the multivariate regression analysis to determine the risk factors associated with the development of adenomatous polyps. We performed a* post hoc* analysis wherein we tested for interaction between* H. pylori* treatment and chronic PPI use in reducing risk for adenomatous polyps in* H. pylori*-infected subjects.

## 3. Results

### 3.1. Cohort Characteristics

There were 943 patients included in the study. The mean age of the cohort was 57 years with 66% of Hispanic origin. Fifty-nine percent of the patients were females. All patients had complete colonoscopies. Sixty-seven percent had chronic PPI use.* H. pylori* was detected in 52% (*n* = 490) of the cohort; and, of these, more than half (*n* = 282) received treatment. Colonic adenomatous polyps were detected in 23% (*n* = 216) of the patients.

Patients who developed adenomatous polyps tend to be older (63 versus 56 years) and male (53%). The incidence of* H. pylori* infection was higher (59% versus 50%) among patients who had adenomatous polyps and there were less chronic PPI users (61% versus 69%) among them.  Table 1 shows the baseline characteristics of patients who developed or did not develop colonic adenomatous polyps.

### 3.2. Risk Factors for Colonic Adenomatous Polyps

In the multivariate regression analysis (Table 3), the risk for developing colonic adenomatous polyps was increased in patients older than 50 years of age (adjusted OR 2.86, 95% CI 1.88–4.35) and those infected with* H. pylori* (adjusted OR 1.55, 95% CI 1.13–2.12). Males were almost twice likely to develop adenomatous polyps than females (adjusted OR 1.97, 95% CI 1.44–2.69). In contrast, chronic PPI use decreased the odds of adenomatous polyp formation (adjusted OR 0.75, 95% CI 0.54–1.05), although this association was not significant.  Table 2 describes the results of the regression analysis in regards to risk factors in the development of adenomatous polyps.

When the cohort was subdivided by age, the odds for colonic adenomatous polyps in* H. pylori* infection were greater in patients who are older than 50 years of age. A cut-off of 50 years was chosen as this is the recommended age to perform screening colonoscopy. The odds for* H. pylori* infection among patients below 50 years of age were increased by 20%, (adjusted OR 1.2, 95% CI 0.49–2.94) but this association is not statistically significant. In patients older than 50 years of age, the odds increased by 65% and became statistically significant (adjusted OR 1.65, 95% CI 1.18–2.33).

### 3.3. Triple Therapy and Chronic PPI Use and the Risk for Colonic Adenomatous Polyps

Among* H. pylori*-infected patients there is a similar trend in the risk factors associated with adenomatous polyp development. Having an age above 50 years and being male were also noted to significantly increase the odds of adenomatous polyp development (adjusted OR 2.76 and 1.79, respectively). Triple therapy decreased the risk by almost two-thirds (adjusted OR 0.69, 95% CI 0.44–1.07). The risk was also reduced in patients with chronic PPI use (adjusted OR 0.69, 95% CI 0.43–1.09). In patients who received triple therapy and were also on chronic PPI use, the risk decreased to one-fourth compared to those who were never treated, not on chronic PPI use, or both (adjusted OR 0.26, 95% CI 0.10–0.646).

**Figure 1:**
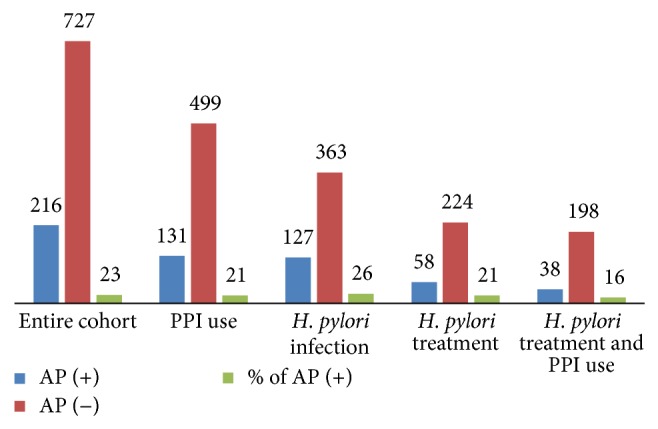
Rates of adenomatous polyps.

Figure 1 shows the summary of the rates of adenomatous polyps in different sample sets in our study: (1) entire cohort (*n* = 943), (2) among chronic PPI users (*n* = 630), (3) among* H. pylori*-infected patients (*n* = 490), (4) among* H. pylori*-infected patients who received triple therapy (*n* = 282), and (5) among* H. pylori*-infected patients who received both triple therapy and were on chronic PPI use (*n* = 236). The rate of adenomatous polyps was highest among* H. pylori*-infected patients (26%) but it went down to 21% in those who received triple therapy and to 16% in those who received triple therapy and were on chronic PPI use.

## 4. Discussion

The purpose of this retrospective case-control study was to answer three questions. The authors want to determine if the presence of* H. pylori* infection correlates with an increased likelihood of colonic adenomatous polyp formation. If the answer to this query was affirmative, the next step was to investigate whether receiving triple therapy ameliorated the risk. Lastly we also wanted to ascertain whether chronic PPI use had an influence on the incidence of adenomatous polyp formation.

Our study had four significant findings:

1. * H. pylori* infection was significantly associated with increased risk of colonic adenomatous polyps, especially in patients older than 50 years of age.
2. Among noninfected patients, chronic PPI use does not reduce the risk of colonic adenomatous polyp formation.
3. Triple therapy or chronic PPI use alone does not significantly ameliorate the risk for colonic adenomatous polyps.
4. The combination of triple therapy and chronic PPI use significantly reduces that risk for colonic adenomatous polyp formation.

In the study that involved the biggest cohort of 156,000 endoscopy results, Sonnenberg and Genta found that* H. pylori* gastritis increased the odds of developing an adenoma by 1.52 times compared to those without* H. pylori* infection [11]. Their cohort had similar characteristics as ours: mean age of 57.8 and being female-predominant. Similarly, our data revealed an increased incidence of colonic adenomatous polyps in the presence of* H. pylori* infection (OR 1.55, *p* value 0.022).

Many patients with* H. pylori* infection are also chronic PPI users. Among those not infected with* H. pylori*, our data did not reveal any statistically significant difference in the risk of polyp formation among chronic PPI users and nonusers (OR 0.92, 95% CI 0.57–1.50). However, when the analysis was limited to only* H. pylori-*infected patients, there is note of a tendency for reduction in risk for adenomatous polyp development with chronic PPI use (adjusted OR 0.69, 95% CI 0.43–1.09). This 30% risk reduction was not seen in two studies that explored an association between PPI use and colon adenomas [13, 14].

The incidence of colonic adenomatous polyp was less in subjects who received triple therapy for* H. pylori*, supporting the hypothesis of previous studies that* H. pylori* might have an influence in the development of colonic polyps. With regard to* H. pylori* and gastric cancers, studies suggest that treatment of the infection reduces the risk of gastric cancer development by 35% [16, 17]. Our analysis suggested a 70% reduction in odds of adenomatous polyp development after adjusting for covariates (adjusted OR 0.69, 95% CI 0.44–1.07). Yet, no study in our review of existing literature has addressed this relationship.

Finally, we determined whether chronic PPI use and* H. pylori* treatment may have an additive effect in the reduction of risk for development of adenomatous polyps in* H. pylori*-infected patients. After testing for interaction, the odds for adenomatous polyps among patients who received triple therapy and were on chronic PPI use decreased to 0.26 (95% CI 0.10–0.646) suggesting that prolonged PPI use and antibiotic therapy for* H. pylori* may reduce the risk of developing colonic adenomatous polyps.

The internal validity of our study may be reduced by the fact that certain covariates were not included in the analysis.* H. pylori* carcinogenesis in the stomach, for example, may not be explained by infection alone and other factors may be in play [18–21]. In addition, certain risk factors for adenomatous polyp formation were also not included in the analysis. Obesity and inactive lifestyle are two studied risk factors for adenomatous polyps [22, 23]. Due to a significant loss of data regarding the basal metabolic index (BMI) and social history (smoking, alcohol, and diet), we were not able to include these factors in the analysis. In addition, 66% of our study population was Hispanic and generalizability of our results may be limited to this certain population. Despite these limitations, we believe that our study provides meaningful results to the existing literature. To our knowledge, our study has a sample size second only to the study done by Sonnenberg and Genta [11]. Most importantly, we are able to provide insight about the effect of triple therapy, chronic PPI use, and the combination of both in the risk of adenomatous polyp formation.

In conclusion,* H. pylori* infection correlated with a higher risk of developing colonic adenomatous polyps.* H. pylori*-infected subjects in the older age group (age > 50 years) were more likely to have these polyps than the younger infected population. Most importantly, our data suggest that treatment of the infection and chronic PPI use correlate with a decreased risk of adenomatous polyp among infected individuals, with a further lowering of this risk when these two treatments are combined.

## Figures and Tables

**Table 1:** Baseline characteristics of patients.

	Adenoma positive	Percent	Adenoma negative	Percent	*p* value
	*n* = 216	(%)	*n* = 727	(%)	
Mean age	62.6		55.8		<0.0001
Sex					
Male	114	52	268	36	Ref
Female	102	47	459	63	<0.0002
Race					
Hispanic	132	61	490	67	Ref
African American	41	19	106	14	0.1
Others	43	20	129	18	<0.29
Chronic PPI use					
Yes	131	61	499	68	Ref
No	85	39	228	31	<0.02
*H. pylori* infection					
Yes	127	58.8	363	50	Ref
No	89	41	364	50	<0.022
Triple therapy + chronic PPI	38	30	198	54	Ref
Triple therapy alone	89	70	165	45	<0.00003

**Table 2:** Multivariate analysis of risk factors affecting development of adenomatous polyps.

Multivariate analysis						
	Crude OR	CI		Adjusted OR	CI	
Age ≥ 50	2.74	1.8	4.28	2.86	1.88	4.35
Sex	1.91	1.4	2.63	1.97	1.44	2.69
Chronic PPI use	0.7	0.508	0.98	0.75	0.54	1.05
*H. pylori* infection	1.43	1.04	1.97	1.55	1.13	2.12

**Table 3:** Multivariate analysis of risk factors affecting development of adenomatous polyps in *H. pylori*-positive patients.

Multivariate analysis						
	Crude OR	CI		Adjusted OR	CI	
Age ≥ 50	3.13	1.81	5.65	2.76	1.59	4.77
Sex	1.83	1.19	2.81	1.79	1.17	2.73
Triple therapy	0.52	0.34	0.801	0.69	0.44	1.07
Chronic PPI use	0.484	0.31	0.76	0.69	0.43	1.09
Triple therapy and chronic PPI use	0.36	0.22	0.78	0.26	0.10	0.646
